# Are protective measures against Covid-19 still active in orthodontic practices? A cross-sectional online survey of French orthodontists three years on from the pandemic

**DOI:** 10.1371/journal.pone.0307453

**Published:** 2024-07-19

**Authors:** Astrid Loiseau, Tiphaine Davit-Béal, Damien Brézulier

**Affiliations:** 1 Pôle Odontologie, CHU Rennes, Univ Rennes, Rennes, France; 2 CHU Rennes, Inserm, Centre d’Investigation Clinique de Rennes (CIC 1414), Rennes, France; 3 ISCR UMR 6226, Univ Rennes, Rennes, France; Universidade Federal Fluminense, BRAZIL

## Abstract

**Purpose:**

The Covid-19 epidemic has imposed profound changes on the practice of orthodontics. It was in this anxiety-inducing context that drastic measures were imposed on orthodontists. The main aim of this online survey is to highlight the measures that are still in place in French orthodontic practices three years after the emergence of the pandemic.

**Methods:**

A cross-sectional online survey was distributed to French orthodontists from march to June 2023. The questionnaire, consisting of 32 questions, was divided into five sections covering habits before and after the pandemic, and the feelings of professionals.

**Results:**

In this survey 230 complete answers were recorded. Three years later, the daily pace had returned to its pre-crisis level. Disinfection and aeration times were still present (p < 0.001). Orthodontists maintained and generalized the use of protective glasses (p = 0.17) and visors (p < 0.001). The same was true for the FFP2 mask and its frequency of change, as well as rigorous hand washing. Finally, the dedicated layout of the practices was maintained: protective screen, filtration system, supply of SHA, travel paths, removal of magazines (for all, p < 0.001).

**Conclusion:**

This study shows that the professional practices imposed by the Covid-19 crisis have been adopted by the majority of French orthodontists, and now appear to be anchored in their routine practice.

**Trial registration number:**

opinion n°2023–004, dated 01.25.2023.

## Introduction

Since the beginning of the 21st century several epidemics linked to coronaviruses have spread. Although these viruses are usually found in animals, they can be transmitted to humans: this is known as zoonosis. It was the case with SARS-CoV, MERS-CoV and Monkeypox [1]. SARS-CoV-2 emerged in December 2019 in Wuhan, China. The WHO classified its spread as pandemic in March 2020. It is still very hot news.

The high potential for contamination, the mode of transmission, the diversity and severity of symptoms, and mutations are all factors that make this virus so dangerous. It is estimated that, on average, a person can infect between 1.5 and 2.5 others if no protective measures are taken. Clinical expression ranges from the absence of symptoms to severe, life-threatening pathologies, particularly in the presence of co-morbidities such as heart or respiratory failure, high blood pressure, diabetes, etc [2].

Although protection against viral risks is well known to dental professionals, it has become a major preoccupation for the general public, and one that is strongly supported by the media. SARS-Cov2 has highlighted the urgent need for a more stringent approach to biological risks [3]. In fact, air is the most common route of transmission, particularly bioaerosols (fine biological particles projected by rotating instruments, stagnant in the air). Orthodontics is directly affected by the patient-practitioner promiscuity imposed by care, the number of patients received per day, and the accumulation of so-called "invasive" or aerosolizing procedures. Infectious risk prevention must ensure the safety of both caregivers and patients. This is a legal, regulatory and ethical obligation. It is ensured by Standard Precautions (SP) published by the French regulation agencies and scientific societies such as the French Federation of Orthodontics. They guide practitioners in hand-washing, professional dress, and the management of medical equipment and devices [4]. In addition, a number of international working groups have examined new recommendations to be implemented during the various confinements [5, 6].

Preventing the risk of contamination has forced practitioners to adapt their practices, with a real impact on their day-to-day professional lives. These measures first appeared in orthodontic practices in 2020, and there was no indication that they would still be in place in the medium term. This is the hypothesis tested here. The aim of the study was to compare the means deployed before the pandemic and those still in place three years later to combat the risk of airborne viral contamination.

## Materials and methods

This cross-sectional study is based on an online survey using the LimeSurvey platform. It was carried out to compare measures to combat the risk of airborne contamination before the pandemic and those still in place three years later. It was carried out according to best practices for conducting an online survey [7].

### Analyzed population

The questionnaire was sent out to French orthodontic specialists in two different ways, both of which provided access to a URL: firstly, via an e-mail campaign (based on scientific society directories); secondly, via the Facebook social network, more specifically on groups dedicated to orthodontists. The mailing campaign took place in three stages, approximately 3 weeks apart, from March to June 2023.

To answer, participants were invited to click on the link and accept the privacy policy. Afterwards, they simply validated their answers, which were automatically recorded in the LimeSurvey database.

The platform’s settings were made compliant with the General Data Protection Regulation (GDPR). This involved no recording of IP addresses and no time stamping of participations. Respondents were thus assured of anonymous storage and data protection on the LimeSurvey server. This protocol made it unnecessary to register with the French Data Protection Authority (CNIL: Commission Nationale de l’Informatique et des Libertés). The study was therefore only declared to the data protection officer (DPO) of the University of Rennes. It issued a favorable opinion on January 25, 2023 (opinion no. 2023–004). Data collection took place from March 8, 2023 to June 2, 2023. Data were exported from LimeSurvey as an Excel file for analysis. It should be noted that only one of the authors (DB) had access to this file. All relevant data are within the manuscript and its (S1 Appendix).

### Online survey

#### Design

The survey was preceded by searches on PubMed and Google scholar. The keywords used were "*infectious risk*"; "*covid-19*"; "*orthodontics*"; "*cross contamination*". These searches were used to target the questions and check the relevance of the survey.

Prior to dissemination, and in line with the recommendations for online surveys, the questionnaire underwent two test phases. The first consisted in checking its validity, i.e. its form (access to hypertext link, readability of responses, graphical errors); the second consisted in feedback from a panel of experts from the target population, on both content and form. Their comments were analyzed by the authors and taken into account to improve the questionnaire’s efficiency [8, 9]. Finally, the questions with qualitative answers were used to calculate Cronbach’s alpha, which in this case was 0.55 and could be considered sufficient [10].

#### Structure

The survey consisted of 32 questions addressed to all participants, 4 of which were optional. The questions were grouped into 5 parts.

Part 1 described the sample. Part 2 described clinical habits from three years ago, prior to the pandemic, according to respondents’ personal memories. Part 3 focused on professionals’ feelings since the pandemic. Part 4 repeated the questions from part 2, at the time of the questionnaire, for purposes of comparison. Finally, the 5th section gave practitioners the opportunity to express themselves freely on the subject.

#### Form of questions

The questions were designed to be as explicit and unambiguous as possible, using simple, understandable vocabulary. They were arranged in a logical sequence to make the questionnaire fun to answer, and to prepare for analysis of the results.

Different types of questions were used. A first type of question used the "point and click" technique in the form of "radio buttons", enabling a quick, easy and unambiguous response while avoiding inconsistencies. This technique was used for dichotomous responses, Likert scales (notion of gradation) and questions requiring a single response (place of exercise; daily rhythm). A second type of question involved numerical entries. The third type was made up of multiple-choice questions, grouped questions and open-ended questions to offer the possibility of free response. Questions were then conditioned to avoid aberrations.

### Statistical analysis

Data was collected automatically by the LimeSurvey platform. They were then exported to a Microsoft Excel® spreadsheet. Descriptive statistics were obtained and compared by period: pre- vs. post-pandemic. Statistical analysis was performed using RStudio® software version 2023.06.1+524 (RStudioTeam) in R language version R 4.3.1 (RCore Team). Qualitative data were analyzed using Pearson’s **χ**2 test with Yates continuity correction. The Shapiro-Wilk test and the Q-Q plot were used to determine the normality of the distribution of quantitative data. Group means were compared using Student’s t-test. Internal consistency of the questionnaire was checked by calculation of Cronbach’s alpha. A univariate logistic regression analysis was performed to identify the use of different professional practices according to the period studied. P values ≤ 0.05 were considered statistically significant.

## Results

### Sample description

Between March 8 and June 3, 2023, 337 responses were received. 230 were complete and have been considered for study, the others, partial, have been excluded.

**Fig 1:**
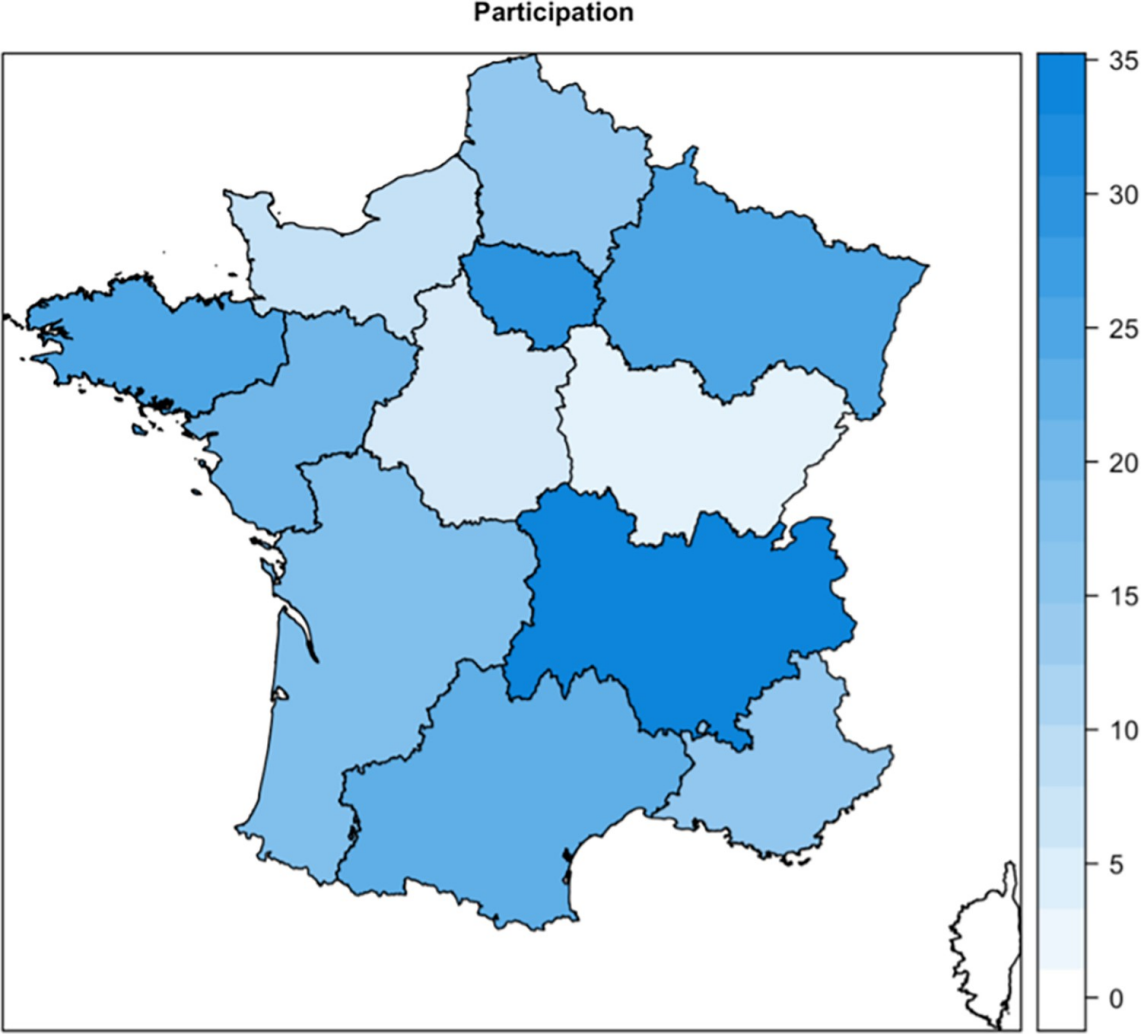
Geographical distribution of participants by French region.

Of the participants, 161 (70%) were women and 69 (30%) men. The mean age of the sample was 44.1 ± 10.9 years. Most had an exclusive orthodontic practice (222; 97%). The geographical distribution of respondents was varied (Fig 1).

### Comparison of attitudes and practices before vs. after Covid-19

#### Daily rhythm and schedule organization

Prior to covid-19, practitioners treated an average of 38.0 ± 13.2 patients per day. For the majority, the volume worked ranged from 7 to 8h/day (86; 37%) to more than 8h/day (118; 51%). After the pandemic, the average number of patients was 37.4 ± 13.2 (p = 0.59) and working hours ranged from 7 to 8h/day (89; 39%) to more than 8h/day (113; 49%) (p > 0.9). There was no difference in these criteria (Table 1).

**Table 1:** Daily rhythm, organization of schedules and, disinfection or aeration times.

	Descriptive analysis		Univariate regression		
	Prior to Covid-19^*1*^	Following Covid-19^*1*^	OR^*2*^^*a2*^	95% CI^*2*^	P-value
Daily patient numbers	38.0 ± 13.2	37.4 ± 13.2	1.00	0.98, 1.01	0.59
Daily working rhythm					0.97
< 5 hours/day	8.0 (3.5%)	8.0 (3.5%)	—	—	
5 to 7 hours/day	18.0 (7.8%)	20.0 (8.7%)	1.11	0.34, 3.62	
7 to 8 hours/day	86.0 (37.4%)	89.0 (38.7%)	1.03	0.37, 2.93	
> 8 hours/day	118.0 (51.3%)	113.0 (49.1%)	0.96	0.34, 2.69	
Schedule organized in time slots					0.075
No	44.0 (19.1%)	30.0 (13.0%)	—	—	
Yes	186.0 (80.9%)	200.0 (87.0%)	1.58	0.96, 2.63	
Time devoted to disinfection					<0.001
No	141.0 (61.3%)	90.0 (39.1%)	—	—	
Yes	89.0 (38.7%)	140.0 (60.9%)	2.46	1.70, 3.60	
Time dedicated to aeration					<0.001
No	175.0 (76.1%)	75.0 (32.6%)	—	—	
Yes	55.0 (23.9%)	155.0 (67.4%)	6.58	4.39, 9.97	

Number of respondents and univariate regression for parameters linked to daily rhythm, organization of schedules and, in particular, use of disinfection or aeration periods.

^1^ Mean ± SD; n (%)

^2^ OR = Odds Ratio, CI = Confidence Interval

Before the pandemic, the majority of practitioners (186; 81%) were already using a schedule organized according to procedures. This organization was even more frequent after the pandemic: 200 (87%) had used it (p = 0.075). As regards the time dedicated to disinfecting the medical room, an inversion of the ratios was highlighted. While only 90 (39%) had scheduled these times before the crisis, 140 (61%) had scheduled them since Covid-19 (p < 0.001). Similarly, only 55 (24%) planned aeration times before the crisis; 155 (66%) have planned them since (p < 0.001) (Table 1).

#### Practice and orthodontist equipment

*Protective glasses and visors*. The majority of doctors wore glasses for clinical procedures (Table 2). After the crisis, the number using eyewear only for aerosol care (48; 21% versus 38; 17%) decreased. The number of practitioners using them for all procedures increased. A total of 134 (58%) wore them systematically before Covid-19, and 157 (68%) afterwards. The number of practitioners not wearing protective eyewear also fell (47; 20%) versus (34; 14.8%). Nevertheless, the study did not reveal any change in the use of protective eyewear between the two periods (p = 0.17).

**Table 2:** Eye protection.

	Descriptive analysis		Univariate regression		
	Prior to Covid-19^1^	Following Covid-19^1^	OR^2^	95% CI^2^	P-value
Use of safety glasses					0.17
I don’t use any	47.0 (20.4%)	34.0 (14.8%)	—	—	
Use for care WITH aerosols	48.0 (20.9%)	38.0 (16.5%)	1.09	0.59, 2.02	
Use for care WITHOUT aerosols	1.0 (0.4%)	1.0 (0.4%)	1.38	0.05, 35.8	
Systematic use for all care	134.0 (58.3%)	157.0 (68.3%)	1.62	0.99, 2.68	
Use of a protective visor					<0.001
I don’t use any	192.0 (83.5%)	112.0 (48.7%)	—	—	
Use for care WITH aerosols	27.0 (11.7%)	93.0 (40.4%)	5.90	3.67, 9.76	
Use for care WITHOUT aerosols	1.0 (0.4%)	1.0 (0.4%)	1.71	0.07, 43.6	
Systematic use for all care	10.0 (4.3%)	24.0 (10.4%)	4.11	1.95, 9.31	

Number of respondents and univariate regression for parameters related to wearing eye protection according to type of care provided.

^1^ Mean ± SD; n (%)

^2^ OR = Odds Ratio, CI = Confidence Interval

In contrast, the use of protective visors increased significantly (p < 0.001). Before the crisis, the majority did not use it (192; 84%), whereas after the crisis only 112 (48%) still did not. For example, 93 practitioners (40%) used it for aerosolized care, compared with 27 (12%) before Covid-19 (Table 2).

*Cap*, *mask and overblouse*. The surgical cap has become an integral part of the orthodontist’s toolkit. Since the crisis, 126 (55%) orthodontists have worn them for both aerosolized and non-aerosolized procedures, compared with 10 (91%) before Covid-19 (Table 3). A large proportion of specialists (90; 39%) now wore it systematically, compared with just 12 (5%) before the pandemic (p < 0.001).

**Table 3:** Surgical caps, surgical masks or FFP2, and overblouses.

	Descriptive analysis		Univariate regression		
	Prior to Covid-19^*1*^	Following Covid-19^*1*^	OR^*2*^	95% CI^*2*^	P-value
Use of surgical mask					<0.001
I don’t use any	17.0 (7.4%)	74.0 (32.2%)	—	—	
Use for care WITH aerosols	16.0 (7.0%)	1.0 (0.4%)	0.01	0.00, 0.08	
Use for care WITHOUT aerosols	5.0 (2.2%)	22.0 (9.6%)	1.01	0.35, 3.35	
Systematic use for all care	192.0 (83.5%)	133.0 (57.8%)	0.16	0.09, 0.28	
Use of a high-filtration FFP2 mask					<0.001
I don’t use any	214.0 (93.0%)	83.0 (36.1%)	—	—	
Use for care WITH aerosols	4.0 (1.7%)	60.0 (26.1%)	38.7	15.3, 130	
Systematic use for all care	12.0 (5.2%)	87.0 (37.8%)	18.7	10.1, 37.6	
Use of protective overblouse or apron					<0.001
I don’t use any	210.0 (91.3%)	134.0 (58.3%)	—	—	
Use for care WITH aerosols	11.0 (4.8%)	80.0 (34.8%)	11.4	6.09, 23.4	
Systematic use for all care	9.0 (3.9%)	16.0 (7.0%)	2.79	1.22, 6.75	
Use of a surgical cap or charlotte					<0.001
I don’t use any	210.0 (91.3%)	104.0 (45.2%)	—	—	
Use for care WITH aerosols	8.0 (3.5%)	36.0 (15.7%)	9.09	4.28, 21.7	
Systematic use for all care	12.0 (5.2%)	90.0 (39.1%)	15.1	8.22, 30.3	
Wearing a mask in the office					<0.001
I put it on as soon as I enter the office	48.0 (20.9%)	192.0 (83.5%)	—	—	
I only wear it during chair care	182.0 (79.1%)	38.0 (16.5%)	0.05	0.03, 0.08	
Frequency of mask change					<0.001
I keep the same one all day long (except in case of soiling).)	44.0 (19.1%)	20.0 (8.7%)	—	—	
I change it once a day (at lunchtime, for example, unless it’s soiled).	118.0 (51.3%)	112.0 (48.7%)	2.09	1.17, 3.82	
I change it 2 or more times a day (and in case of soiling).	56.0 (24.3%)	92.0 (40.0%)	3.61	1.96, 6.86	
I change it between each patient	12.0 (5.2%)	6.0 (2.6%)	1.10	0.34, 3.27	

Number of respondents and univariate regression for parameters related to the use of surgical caps, surgical masks or FFP2, and overblouses.

^1^ Mean ± SD; n (%)

^2^ OR = Odds Ratio, CI = Confidence Interval

Before the pandemic crisis, the FFP2 mask was hardly ever used (214; 93%). After the pandemic, only 83 (36%) practitioners did not use them. For 87 (38%) practitioners, it became systematically worn for all care. Wearing a surgical mask on entering the practice had become routine for 192 (84%) practitioners after the crisis. This was the case for only 48 clinicians (21%) before the pandemic. In fact, only 38 practitioners (17%) used a mask only for chairside care since Covid-19 (p < 0.001). The mask was also changed more frequently. A total of 44 (19%) kept the same mask throughout the day, versus 20 (9%) at present. While the majority continued to change their masks in the middle of the day 118 (51%) versus 112 (49%) after the pandemic, more of them changed their masks more than twice a day (56; 24% vs. 92; 40%) (Table 3).

The majority of practitioners did not use a protective apron (or overblouse) either before (210; 91%) or after covid-19 (134; 58%). However, there was a clear increase in its use (p < 0.001). During procedures involving the risk of aerosolization, 11 practitioners (5%) used it before Covid-19, compared with 80 now (35%) (Table 3).

*Hand washing and glove wearing*. With regard to hand hygiene, only 35 (15%) practitioners had no hand-washing routine, compared with 57 (25%) before the crisis. The majority used hydroalcoholic gel friction between each patient, both before (109; 47%) and after the pandemic (127; 55%). This showed a slight improvement in hand hygiene practices (p = 0.046) (Table 4).

**Table 4:** Hand hygiene and glove wearing.

	Descriptive analysis		Univariate regression		
	Prior to Covid-19^*1*^	Following Covid-19^*1*^	OR^*2*^	95% CI^*2*^	P-value
Use of gloves					0.91
I don’t use any	3.0 (1.3%)	2.0 (0.9%)	—	—	
Use for care WITH aerosols	1.0 (0.4%)	2.0 (0.9%)	3.00	0.16, 97.6	
Use for care WITHOUT aerosols	1.0 (0.4%)	1.0 (0.4%)	1.50	0.04, 57.1	
Systematic use for all care	225.0 (97.8%)	225.0 (97.8%)	1.50	0.25, 11.5	
Hand-washing and hygiene					0.046
I don’t have a hand-washing routine	57.0 (24.8%)	35.0 (15.2%)	—	—	
I systematically wash my hands with mild soap between each patient	53.0 (23.0%)	51.0 (22.2%)	1.57	0.89, 2.78	
I systematically wash my hands with mild soap and then with hydroalcoholic friction between each patient.	11.0 (4.8%)	17.0 (7.4%)	2.52	1.07, 6.14	
I systematically wash my hands with hydro-alcoholic friction between each patient.	109.0 (47.4%)	127.0 (55.2%)	1.90	1.16, 3.13	

Number of respondents and univariate regression for parameters related to hand hygiene and glove wearing.

^1^ Mean ± SD; n (%)

^2^ OR = Odds Ratio, CI = Confidence Interval

The vast majority of practitioners used gloves for every clinical procedure. No change in glove use was observed (225; 97.8% vs. 225; 97.8%) (p = 0.91).

#### Cabinet fittings

Since the pandemic, travel paths have been frequently implemented: 97 (42%) versus 44 (19%) previously (p < 0.001). Protective screens were introduced after the crisis in 161 practices (70%) versus 14 (6%) initially (p < 0.001). Practitioners also reviewed the air filtration systems in their practices (35; 15.2% vs. 89; 38.7%) (p < 0.001). Magazines in the waiting room disappeared, with only 14 (6%) practices retaining them (p < 0.001).

The use of preoperative mouthwash was imposed on patients (17; 7% vs. 34; 15%) and hydroalcoholic solution was made available to them (65; 28% vs. 220; 96%) (p < 0.001) (Table 5).

**Table 5:** Orthodontic office layout parameters.

	Descriptive analysis		Univariate regression		
	Prior to Covid-19^*1*^	Following Covid-19^*1*^	OR^*2*^^*a51*^	95% CI^*2*^	P-value
Protective screen (plexiglass type)					<0.001
No	216.0 (93.9%)	69.0 (30.0%)	—	—	
Yes	14.0 (6.1%)	161.0 (70.0%)	36.0	20.2, 68.9	
Air filtration system					<0.001
No	195.0 (84.8%)	141.0 (61.3%)	—	—	
Yes	35.0 (15.2%)	89.0 (38.7%)	3.52	2.27, 5.55	
Provision of hydroalcoholic solution for patients					<0.001
No	165.0 (71.7%)	10.0 (4.3%)	—	—	
Yes	65.0 (28.3%)	220.0 (95.7%)	55.8	29.2, 119	
Systematic preoperative mouthwash					0.011
No	213.0 (92.6%)	196.0 (85.2%)	—	—	
Yes	17.0 (7.4%)	34.0 (14.8%)	2.17	1.19, 4.10	
Travel paths					<0.001
No	186.0 (80.9%)	133.0 (57.8%)	—	—	
Yes	44.0 (19.1%)	97.0 (42.2%)	3.08	2.04, 4.72	
Magazines available in the waiting room					<0.001
No	33.0 (14.3%)	216.0 (93.9%)	—	—	
Yes	197.0 (85.7%)	14.0 (6.1%)	0.01	0.01, 0.02	
Professional cleaning company for clinical clothing					0.63
No	187.0 (81.3%)	191.0 (83.0%)	—	—	
Yes	43.0 (18.7%)	39.0 (17.0%)	0.89	0.55, 1.43	
In-office washing machine for clinical clothing					<0.001
No	137.0 (59.6%)	81.0 (35.2%)	—	—	
Yes	93.0 (40.4%)	149.0 (64.8%)	2.71	1.86, 3.97	

Number of respondents and univariate regression for orthodontic practice layout parameters.

^1^ Mean ± SD; n (%)

^2^ OR = Odds Ratio, CI = Confidence Interval

Cleaning of professional clothing was initially carried out within the practice. In fact, only (43; 18.7%) of practitioners had recourse to an external service provider. This figure was maintained after Covid-19 (p = 0.63). However, the number of practitioners equipped with a washing machine increased between the two periods (93; 40% vs. 149; 65%) (p < 0.001).

### Perception of the risk of viral transmission and cross-infection

A series of questions dealt with the perceived risk of viral transmission within the orthodontic practice. The majority of practitioners agreed that the risk was high for them (196; 85%) and their assistants (117; 51%), even when treating children or adolescents. For a majority, however, the risk seemed more limited in orthodontics than in general practice (158; 68%) (Table 6).

**Table 6:** Respondents’ perception of the risk of cross-contamination.

Risk of cross-contamination	No, not at all ^*1*^	Yes, it is ^*1*^	Rather no ^*1*^	Rather yes ^*1*^	No opinion ^*1*^
Lower for the assistant	69 (30%)	26 (11%)	48 (21%)	78 (34%)	9 (3.9%)
Lower when caring for children	128 (56%)	3 (1.3%)	62 (27%)	16 (7.0%)	21 (9.1%)
Lower than in general practice	32 (14%)	65 (28%)	32 (14%)	93 (40%)	8 (3.5%)
High for the orthodontist	2 (0.9%)	133 (58%)	24 (10%)	63 (27%)	8 (3.5%)

Respondents’ perception of the risk of cross-contamination in the orthodontic practice.

^1^ n (%)

Although specialists considered the risk of cross-contamination to be high, a higher state of anxiety was not evidenced. This may be explained by the greater attention paid to this risk since the pandemic. On a scale of 1 ("no, I’m not more alert to the risk of cross-contamination since Covid-19") to 5 ("yes, I’m extremely more alert to the risk of cross-contamination since Covid-19"), orthodontists rated their awareness at 3.8 ± 1.2.

More specifically, feelings of anxiety were not described for aerosolized care (171, 75%), check-ups or explanations (214, 93%), non-aerosolized care (216, 89%) or invasive procedures such as mini-screw insertion (180, 78%).

### Impact of viral infection risk on orthodontic practices

Thirdly, it seemed relevant to assess how the pandemic peak had impacted orthodontic technical practices. It emerged that the rate of patients undergoing orthopedic treatment changed little between the two periods, 27.2 ± 12.1% and 27.5 ± 13.7% respectively before and after the peak (p = 0.77). The trend was similar for braces: 62.5 ± 15.1% and 60.1 ± 17.7 before and after (p = 0.13). Finally, the number of patients treated with aligners rose slightly from 9.7 ± 10.4% to 11.6 ± 12.0%, without being significant (p = 0.071). Finally, the number of practitioners using remote monitoring remained low, at around 10% of respondents whatever the period studied.

## Discussion

The Covid-19 pandemic hit France in March 2020. Since then, orthodontists, following the recommendations of various societies, have modified their professional practices [11]. Preventing the risk of contamination has become a challenge that has forced us to adapt our practices, with a real impact on thousands of healthcare professionals [12]. These changes have taken place in an extremely anxiety-provoking context. Moreover, they have sometimes illustrated the absence of consultation or real proof of effectiveness [13]. Three years after this episode, it seemed appropriate to study the protective measures that have endured in orthodontic practices, and how French orthodontists feel about them.

The online survey received 337 responses, 230 of which were complete and usable. Over 10% of French orthodontists responded, making the survey representative of the population. The distribution was homogeneous across practice regions. However, it is likely that the management of the risk of contamination differed according to whether the practice was rural or urban. In fact, it has already been shown, in connection with Covid-19, that the place of practice influenced practices [14]. Finally, to keep the questionnaire as simple as possible, the type of professional structure was not entered, even though it could have had an impact on the responses. Also, this type of online survey is known to have several weaknesses. We are well aware that orthodontists with little sensitivity to this issue may not have responded, which focuses the results on the part of the population that has potentially taken the most precautions. With regard to pre-pandemic practices, a memory bias is quite conceivable, and seems difficult to avoid.

Among orthodontists, the majority considered the risk of cross-contamination for themselves and their team to be high. The risk appeared similar for the practitioner and his assistant, whether treating children or adults. The risk was considered lower than for general dentists. The lower number of aerosolizing procedures could explain this, even if the frequency of orthodontic consultations received daily is higher.

The risk of contamination was no longer a source of anxiety as it had been in the past. This feeling did not last, although it was strongly reported during the pandemic peaks of 2020 [15]. At the time, some practitioners went so far as to say that they had stopped working.

Three years after the pandemic, it appeared that French orthodontists had resumed a daily rhythm quite comparable to that prior to the crisis. Opening hours were in the region of 7 to 8 hours a day, enabling around forty patients to be seen. However, a survey carried out at the end of the crisis showed that most practitioners wanted to increase the number of hours they worked per week, while at the same time reducing the number of patients they saw each day [16]. It’s a safe bet that the organizational constraints raised by such changes prevailed over their implementation.

While the pace of work has not changed, schedule management has. Time slots have been dedicated to disinfection. Room ventilation was also reinforced. Contamination-risk procedures, for example, could be performed in a dedicated room. This is the case, for example, for the removal of equipment [17]. These changes in practice were in line with French professional recommendations. Over and above organizational considerations, specific features were implemented and maintained in the practices. Although the French Health Authority (HAS: Haute Autorité de Santé) has not made any specific recommendations on the flow of people within practices, practitioners have adapted their offices on a permanent basis where possible, for example by introducing travel paths. Others maintained improved filtration-ventilation systems, or increased the availability of hydroalcoholic solutions for patients. Finally, it should be noted that a significant number of orthodontists imposed a mouthwash before any surgical procedure. It has been shown to significantly reduce microbial load, especially povidone-iodine, which is particularly effective against respiratory viruses [18, 19]. Nevertheless, a recent systematic review points to the discrepancy in results and concludes that the effectiveness of different mouth rinses to reduce viral infectivity, improve clinical symptoms or prevent SARS-CoV-2 infection remains inconclusive [20].

Nonetheless, most of the changes that have taken place over the past 3 years relate to personal protective equipment: masks, gloves, hoods and eyewear. Although orthodontists initially wore protective glasses (particularly for invasive procedures involving the risk of splashing or aerosolization), they have now systematically adopted their use. The trend is the same for protective visors. However, the literature is contradictory on their effectiveness in view of the risk of contamination by aerosolization [21]. However, they are useful for protecting against eye injuries caused by archwires, ligatures or other products (etching, adhesive, cement) [22]. The medical team should be encouraged to wear eye protection [4].

The second major change concerned the mask. This device is perfectly effective in protecting against contamination by droplets or aerosols [23]. In the past, orthodontists did not systematically wear a mask during care compared to general dentists [24]. Three years after the crisis, over a third of orthodontists surveyed now wear them systematically. The crisis has led to the introduction of FFP2 masks to filter out the virus. Surgical or FFP2 masks were now worn throughout the office, regardless of the activity performed. On average, they were changed twice a day. This goes against the recommendations; the CDC recommends changing masks between patients. Practitioners did, however, change their masks when they became soiled.

The third key change concerned hand hygiene. This is the most important step in the fight against infections [25]. The literature shows that transmission of many respiratory infections can be controlled by handwashing with soap or rubbing with hydroalcoholic solution [26]. It turns out that, since the crisis, orthodontists have adopted the practice of performing a hydroalcoholic rub between each patient, which was not previously the case. This is also in line with WHO recommendations, which state that washing with soap is recommended at the beginning and end of the day, or when hands are visibly soiled [27]. Gloves were also worn for hand hygiene. However, gloves have a high rate of perforations, sometimes imperceptible, mainly during orthodontic procedures. For example, more than half of them show perforations during procedures on fixed appliances [28].

When asked about orthodontic techniques and practices, professionals replied that they had not changed them since the crisis. The ratio of patients treated with braces, functional appliances and aligners remained similar. Remote monitoring, which would optimize the number of appointments and therefore the risk of contamination, had not been developed [29, 30]. Similarly, the rate of treatment with aligners has not been affected by the crisis. Yet this type of treatment has a much lower projection risk than conventional fixed treatments, and has already been shown to generate a low number of potential emergency appointments [31].

## Conclusion

Three years after the SARS-CoV-2 pandemic crisis, which had a profound impact on healthcare professionals, it seemed appropriate to evaluate the professional practices and arrangements deployed since then and in an anxiety-inducing context, and which have been perpetuated in orthodontic offices. The study showed that these changes concerned both the medical team and patients. Taken together, these measures have helped bring orthodontists’ anxiety levels back to pre-2020 levels. The main changes still in place in orthodontic practices were of two types:

- Practice management and organization: dedicated time for bio-cleaning, people flow, provision of hydroalcoholic solution;
- Wearing of personal protective equipment: mask, glasses, and hand hygiene.

## Supporting information

S1 Appendix Raw data from questionnaires sent to practitioners. (XLSX)
